# Improving Pediatric/Neonatology Residents' Newborn Resuscitation Skills With a Digital Serious Game: DIANA

**DOI:** 10.3389/fped.2022.842302

**Published:** 2022-04-01

**Authors:** Serena Bardelli, Giulio Del Corso, Massimiliano Ciantelli, Marta Del Pistoia, Francesca Lorenzoni, Nicoletta Fossati, Rosa T. Scaramuzzo, Armando Cuttano

**Affiliations:** ^1^Centro di Formazione e Simulazione Neonatale “NINA,” U.O. Neonatologia, Dipartimento Materno-Infantile, AOUP, Pisa, Italy; ^2^Department of Mathematics, Gran Sasso Science Institute (GSSI), L'Aquila, Italy; ^3^U.O. Neonatologia, Dipartimento Materno-Infantile, AOUP, Pisa, Italy; ^4^Institute of Medical and Biomedical Education, Faculty of Medicine, St. George's University of London, London, United Kingdom

**Keywords:** DGBL, digital games, technology-enhanced training or learning, neonatal resuscitation, memory and retention, newborn infants, healthcare education, serious game

## Abstract

**Background:**

Serious games, and especially digital game based learning (DGBL) methodologies, have the potential to strengthen classic learning methodology in all medical procedures characterized by a flowchart (e.g., neonatal resuscitation algorithm). However, few studies have compared short- and long-term knowledge retention in DGBL methodologies with a control group undergoing specialist training led by experienced operators. In particular, resident doctors' learning still has limited representation in simulation-based education literature.

**Objective:**

A serious computer game DIANA (**DI**gital **A**pplication in **N**ewborn **A**ssessment) was developed, according to newborn resuscitation algorithm, to train pediatric/neonatology residents in neonatal resuscitation algorithm knowledge and implementation (from procedure knowledge to ventilation/chest compressions rate). We analyzed user learning curves after each session and compared knowledge retention against a classic theoretical teaching session.

**Methods:**

Pediatric/neonatology residents of the Azienda Ospedaliera Universitaria Pisana (AOUP) were invited to take part in the study and were split into a game group or a control group; both groups were homogeneous in terms of previous training and baseline scores. The control group attended a classic 80 min teaching session with a neonatal trainer, while game group participants played four 20 min sessions over four different days. Three written tests (pre/immediately post-training and at 28 days) were used to evaluate and compare the two groups' performances.

**Results:**

Forty-eight pediatric/neonatology residents participated in the study. While classic training by a neonatal trainer demonstrated an excellent effectiveness in short/long-term knowledge retention, DGBL methodology proved to be equivalent or better. Furthermore, after each game session, DGBL score improved for both procedure knowledge and ventilation/chest compressions rate.

**Conclusions:**

In this study, DGBL was as effective as classic specialist training for neonatal resuscitation in terms of both algorithm memorization and knowledge retention. User appreciation for the methodology and ease of administration, including remotely, support the use of DGBL methodologies for pediatric/neonatology residents education.

## Introduction

Globally, an estimated 2.5 million newborns die each year worldwide from childbirth asphyxia (defined as a failure to initiate or sustain spontaneous breathing at birth) (1) as ~15% of full term births require effective resuscitation (2). Correctly performed neonatal resuscitation can save around 700,000 lives worldwide every year (SIN [Società Italiana di Neonatologia, Italian Neonatology Society] Survey on the organization of care in the delivery room, 2020). However, resuscitation guidelines are not adhered in more than 90% of cases (3).

Digital game based learning (DGBL) methodologies have proved effective in multiple medical contexts (4–6) by integrating the advantages of the classic teaching process with the possibilities offered by the use of simulations (replicability, standardized teaching environment, user adaptability of the procedure). They can be applied to most flowchart-based medical procedures and, crucially, their high repeatability and the possibility of dividing each session into several parts can stimulate procedural memory (7, 8). Further advantages of DGBL methodologies include the provision of an optimal context for user result analysis (every action performed by the learner is stored) and a higher attention/appreciation rate by users.

While it is questioned whether DGBL approach can fully replace classic teaching methodologies (9–12), DGBL methods are known to be effective in checking what was learned and reinforcing motivation to enhance adult learning in medical education (13) and, more in general, in higher education (14). With particular regard to medical practice (14–16), and especially neonatal resuscitation (9, 17, 18), numerous existing studies demonstrate the effectiveness of DGBL/simulation methods in stimulating better learning. However, many of these studies lack a scoring baseline (pre-test), a subsequent follow up to evaluate knowledge retention, and/or a homogeneous and independent control group.

DGBL methodologies can be applied to most flowchart-based medical procedures. In this study, we implemented a new ad hoc digital serious game **DIANA** (**DI**gital **A**pplication in **N**ewborn **A**ssessment) and we developed it for neonatal resuscitation teaching. Rather than focusing on a single skill (e.g., endotracheal intubation) this computer game aims to teach the entire neonatal resuscitation algorithm. Unlike most published studies, which involved medical students (9, 19, 20) and expert neonatal professionals (17, 21) as learners, we tested it on a group of resident students of varying experience, using a randomized control study design with the primary goal of testing short- and medium-/long-term knowledge retention [primary endpoint: compare knowledge retention of DGBL and classical training]. The analysis is done by comparing the DGBL group with an independent group undergoing classic training (e.g., 80 min theoretical teaching session provided by an expert neonatal trainer). Indeed, despite an autonomous training using didactic material (9), the choice of a guided approach provides a more controlled training path (10). In addition, several other secondary endpoints were tested to evaluate the performance obtain from DGBL recording scores: knowledge scores, time decision, ventilation/chest compression rate, and user acceptance of this new training methodology.

## Materials and Methods

### Software Description

**Figure 1:**
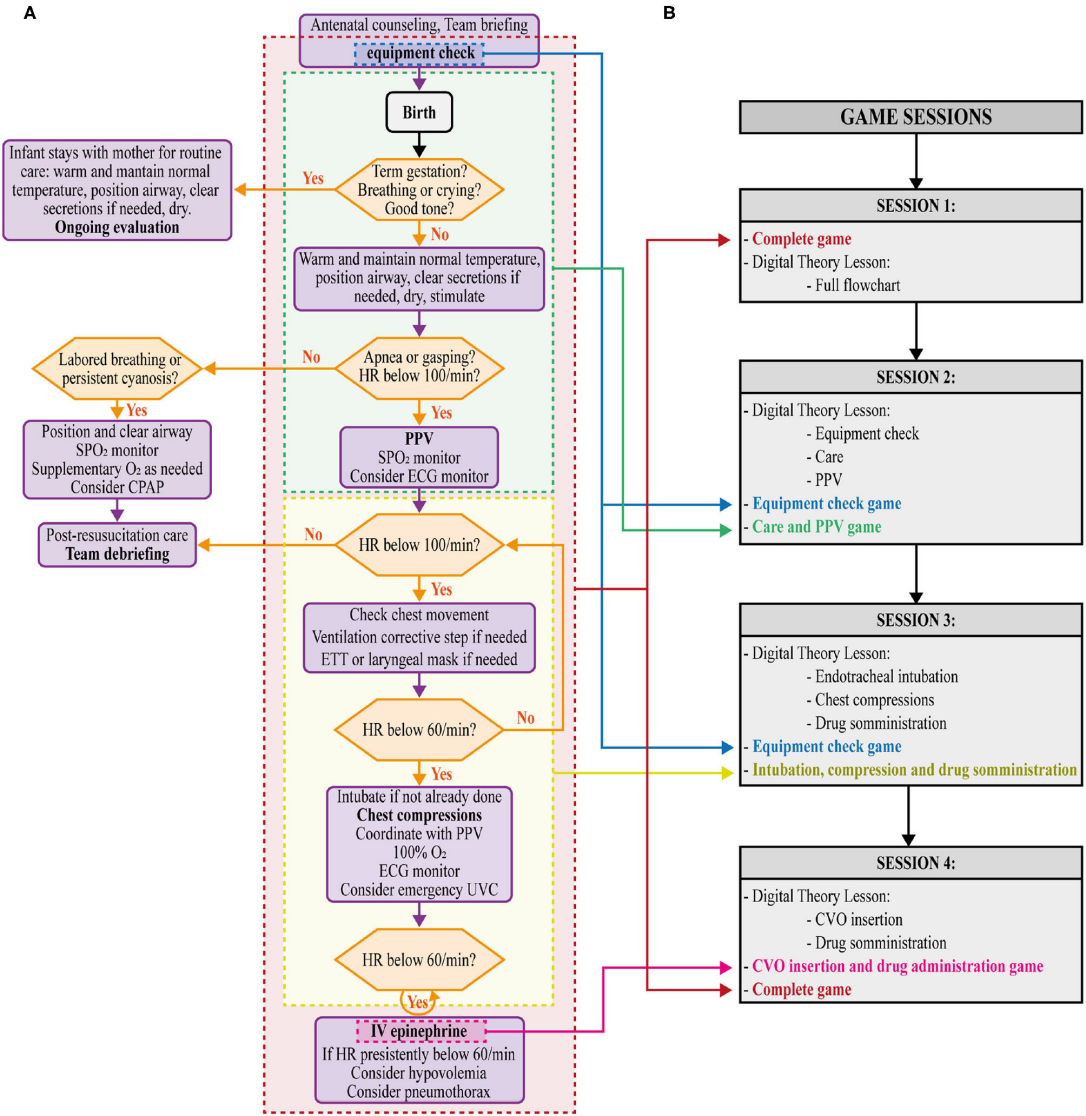
**(A)** Newborn resuscitation flow chart showing corresponding sections in the DIgital Application in Newborn Assessment (DIANA) game (equipment check, neonatal care and PPV, intubation, chest compression and drug administration, umbilical vein catheterization [UVC, CVO in Italian and consequently in this game version] and drug administration, and complete). **(B)** Details of the game sessions (1,2,3,4).

The DIANA software was developed according to newborn resuscitation flowchart to verify DGBL methodology for training. The DIANA software code was implemented with the real-time development platform Unity (https://unity.com/). The video game was divided into four sessions (i.e., distributed study) with an inter-study interval (ISI) of 48 h to consolidate information memory through repetition (4). Each game session consisted of a theoretical and an interactive part. The interactive part started with 1 min of equipment check. The interactive part simulated a clinical case, where the user would choose how to proceed from one of four options provided. A virtual assistant would intervene in case of errors, and provide detailed instructions to enhance learning without diminishing the gaming experience (22). In the theoretical part, the same virtual assistant, with a human voice, would give a theoretical tutorial using videos to demonstrate technical skills. The first session included an interactive game and complete theoretical teaching about the whole neonatal resuscitation procedure. In the second session, the theoretical part addressed equipment check, neonatal care, and assisted ventilation. The interactive part of the video game followed on from the first session, with successful resuscitation after correctly assisted ventilation. In the third session, the theoretical part dealt with endotracheal intubation skills, chest compressions, and drug delivery, with the interactive part of the video game ending after the execution of chest compressions. Lastly, the fourth session consisted of three activities: a tutorial on venous umbilical catheter insertion, a mini game related to the procedure, and the full execution of resuscitation simulation as in the first session (Figure 1). To the aim of the present study, residents did not have free access to the software except for sessions scheduled on the basis of the time intervals described in the study.

In this work, we scheduled the DIANA sessions to ensure the same time practice between residents. However, for future practical uses of DIANA to support classical training, this fixed schedule is not imposed by the software. Indeed, DIANA does not impose on the user the sequential use of the game levels (e.g., a practitioner can freely select one of the four sessions). This allows the end user to freely practice on a single flowchart topic or to assess their knowledge of the entire algorithm. The only limitation is that the user within the session will be guided to follow the theoretical part first and then the practical part.

**Figure 2:**
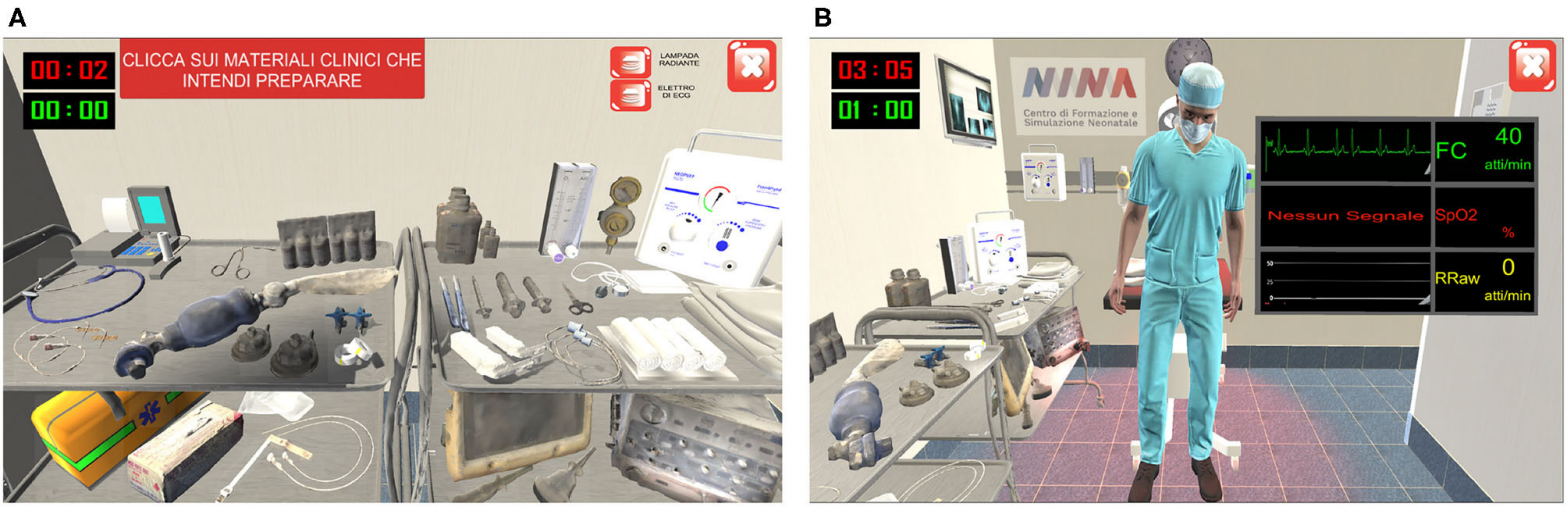
Software screenshots: Equipment check **(A)** and dynamic curves of the simulated newborn's main vital signs **(B)**. In equipment check **(A)**, the user follow the instruction of the game in the red box in the left corner (in English: “click on the materials you want to check”).

**Figure 3:**
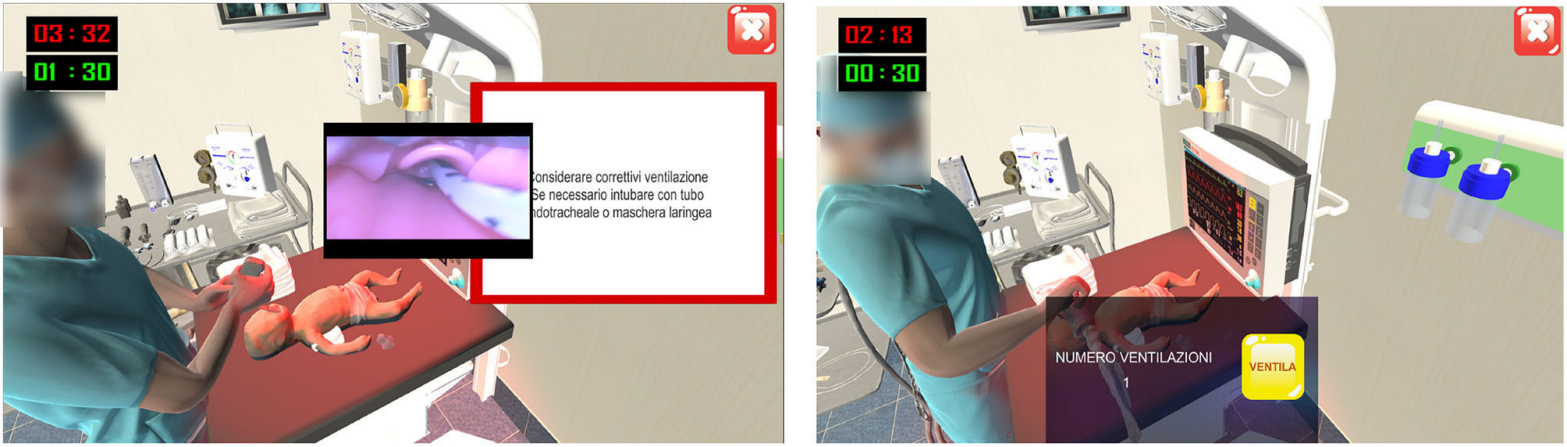
Software screenshots: Execution of endotracheal intubation and assisted ventilations by the virtual assistant. During the execution by virtual assistant, the user gets some useful advice as seen in the white panel: “consider corrective actions for ventilation, such as endotracheal intubation or laryngeal mask insertion.” During the execution of assisted ventilation, the virtual assistant squeezes the Ambu bag when the users click on button VENTILA (“ventilate”). The number of ventilation acts performed is showed next to VENTILA button.

Within the interactive video game, the user had 1 min to select the essential tools (Figure 2A), categorized as totally correct, partially correct, and incorrect. Depending on the tool, size and setting selection would be required. After 1 min, the chosen tools would appear in a box, checked in green (“selection made”) or red (“missing” equipment). When assessing the clinical state of the patient, a monitor would show dynamic curves and heart rate, respiratory rate, and oxygen saturation (Figure 2B). Practical procedures were performed by the virtual assistant (Figure 3). During ventilation execution, the user defined the timing of the ventilation by selecting a “Ventilation” button. The game was designed to last 30 s, during which, every 10 s, the assistant's voice would reassuringly provide feedback to the user, e.g., advising them to increase or reduce the rhythm or complimenting him/her for maintaining an optimal respiratory rate in assisted ventilation. Importantly, chest compressions execution would imply cooperation between user and virtual assistant: the former would perform the required three chest compressions, following one assisted breath by the latter.

### Study Design and Procedure

**Figure 4:**
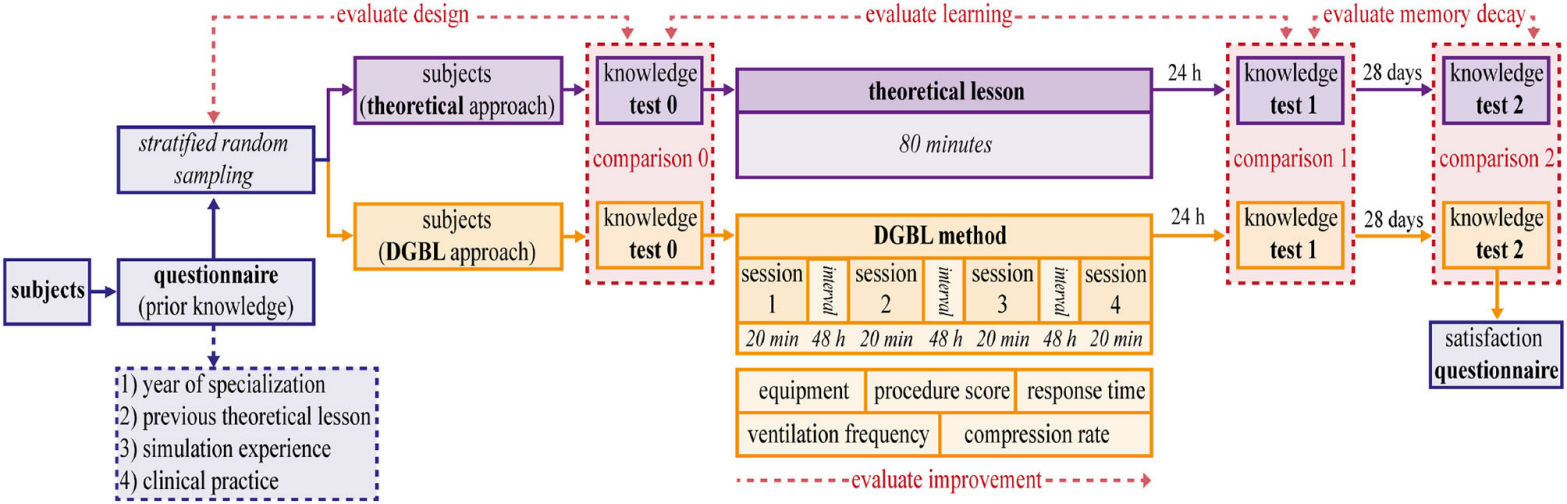
Study analysis scheme. Subjects are divided using a stratified random sampling into two homogeneous and independent groups, based on the score in a prior knowledge questionnaire. The first group (theoretical lesson) is trained by an expert neonatal trainer for 80 min. The second group (digital game based learning [DGBL] method) is trained using DIgital Application in Newborn Assessment (DIANA) for the same length of time on four different sessions. Three written tests (0 pre-test, 1 post-test, 2 follow-up) are used to compare the methodologies (comparisons 0, 1, 2) and to evaluate learning and memory decay. The knowledge test 0 is used to evaluate the stratified random sampling.

Study participants filled a questionnaire to assess their previous knowledge and experience (Figure 4). Based on questionnaire results, two homogeneous groups [Stratified random sampling, similar to other DGBL studies, (12, 23)] were randomized to either the classic teaching process (frontal teaching session) or the one based on digital simulations (DGBL), respectively.

The theoretical teaching session (Figure 4, in purple) was given in person by an expert neonatal trainer, with no more than 10 medical residents for each group, which allowed them to take a very interactive lesson. After finishing the theoretical part, residents practiced the technical skills of PPV, chest compression, and endotracheal intubation on a medium fidelity mannequin (Newborn ANNE, https://laerdal.com/it/doc/222/Newborn-Anne). Neither in the theory lesson nor in the software a specific (limited) clinical case was presented and discussed. On the contrary, in both training residents were asked to perform the whole resuscitation algorithm.

The DGBL group training methodology is based on the use of DIANA software. The software guided the user through the entire resuscitation flowchart divided into four phases. Indeed, starting from the promising results obtained even with a single session of a serious game approach (9, 17, 21), DGBL group (Figure 4, in orange) training was based on the natural subdivision allowed by a digital game: four sessions of 20 min each, separated by a 48 h break; knowledge tests began 24 h after the last session, with the same evaluation process as for the classic training group.

Both the groups (Figure 4, in purple) underwent three knowledge tests about neonatal resuscitation algorithm and equipment check. The test was administered at three different times: immediately before the tutorial (pre-test 0), at 24 h (post-test 1) and at 28 days (follow-up test 2) after training ending; the questions and answers remained the same, while their order was randomly altered. Specific time intervals between assessments were chosen to capture actual knowledge retention. A 24 h post-training time interval was specifically chosen to filter out the positive effects of short-term memory on scores (24). The 28-day interval to evaluate of memory decay has been widely used in DGBL (25). Unlike a much longer interval adopted by other authors (9, 17), it minimizes the high risk of study drop out within a medical resident population, or the confounding effect of further training. Similarly, candidates were not made aware of our study's assessment methods and timings, including the 28-day delayed test, in order to prevent skewed outcomes. The three scores for either learning method were compared to evaluate the two methodologies, their strengths and limitations (comparisons 0, 1, 2 in red in Figure 4). Knowledge test 0 was also used to evaluate the design.

Furthermore, in DGBL group, user improvement was evaluated as the sessions progressed by recording any change in individual tests' numerical values (equipment score, procedure score, response time, ventilation frequency, compression rate) as common in DGBL methodologies (26). At the end of data collection, a user satisfaction questionnaire was administered to DGBL group, to integrate subsequent versions of the software with user suggestions.

### Measures

The primary endpoint of this study is to compare the effectiveness between DGBL (DIANA) and classic learning methodology on knowledge retention based on knowledge questionnaire performance. The several secondary endpoints regarding the evaluation of the effectiveness of the DGBL methodologies on the user's performance during the gaming sessions and the satisfactions evaluation of this new methodology are summarized in Table 1 and described in sections “Knowledge test scores” and “DGBL scores.”

**Table 1:** Description of the variables observed during the study divided between primary endpoints (evaluate the effectiveness of DGBL and classic learning methodology on knowledge retention) and secondary endpoints (evaluate the effectiveness on user's performance during the gaming sessions).

**Comparison**	**Feature observed**	**Comparison Tool**	**Question to answer**
DGBL (DIANA) and classic learning methodology [primary endpoint]	Knowledge retention and equipment checklist	Knowledge tests (pre-training, 1 day post, and 28 days post-training)	Did the DGBL training methodology prove as effective as theoretical teaching session in knowledge retention?
DGBL(DIANA) games performance [secondary endpoints]	Knowledge retention	Performance of different session game scores	Was the DGBL training methodology effective to learn a flowchart reducing decision time and increasing scores results?
	Equipment checklist		Was the DGBL training methodology effective to learn the equipment checklist?
	Ventilation rate		Was the DIANA ventilation game effective to learn the correct ventilation rate to perform during a PPV procedure?
	Chest compression rate		Was the DIANA chest compression game effective to learn the correct rate to perform during a newborn resuscitation?
	Satisfaction of new methodology	Satisfaction questionnaire	Has the DGBL methodology been considered useful and effective by users?

#### Knowledge Test Scores

Knowledge tests are used in DGBL analysis to evaluate performance (20). The test used in this work was written by neonatal resuscitation trainers accredited by SIN, and consisted of 21 questions (each with 1 correct and 5 incorrect answers) related to the correct resuscitation procedure and a list of 40 items (21 correct, 6 partially correct, and 13 incorrect) to check. The knowledge test score was calculated by allocating 1 point for each correct answer, 0 for null, and −0.2 for incorrect ones, so that the average score could be assumed to be zero in case of randomly selected answers. The result was then normalized by the number of questions. The equipment score, on the other hand, consisted of the number of correct instruments (21) selected from the list of 40 items.

#### DGBL Scores

During the execution of DIANA game, the following parameters were recorded: decision-making/response time, answer correctness from the multiple options included in the simulation, choice of equipment before each simulation, uniformity, and correctness of ventilations/compressions timing. A positive score was assigned for a correct answer, a negative value for an incorrect selection, and a neutral (null) score for selecting the “*Get help”* option, available for every question to cover the operator's inability to make a decision. Choosing this option was followed by a detailed explanation of the correct decision by the virtual assistant to stimulate learning and improve subsequent sessions' performance. Knowledge score was calculated as the number of correct answers normalized by the number of questions for each session. The equipment score consisted of the number of correct instruments selected from a list of 40 items (21 correct, 6 partially correct, and 13 incorrect). As some game sessions covered only part of the resuscitation procedure (Figure 1), the knowledge score was calculated on three question subsets: on care and assisted ventilation (PPV) (sessions 1-2-3-4), on intubation and compressions (sessions 1-3-4), and those on drug administration (session 1-4), respectively. For each answer, the response time (i.e., the time elapsed between the question administration and the execution of the action) was also calculated.

#### Compression and Ventilation Scores

**Figure 5:**
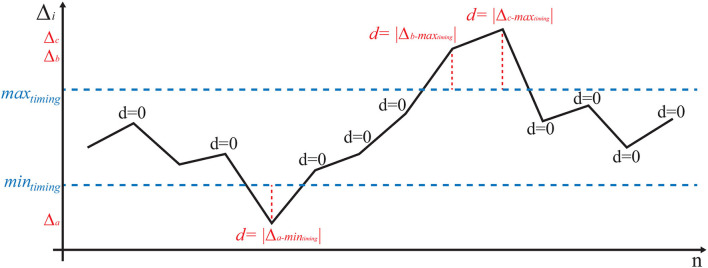
Example of a possible of ventilation/compression pattern (black). If the Δ_*i*_ between two consecutive acts is correct, it falls between the horizontal dashed lines *y* = *min*_*timing*_ and *y* = *max*_*timing*_; in this case, the value is considered perfectly correct (e.g., *d* = 0). Excessively irregular patterns lead to a positive value of *d* (red).


In the games involving compressions and ventilations, choosing a score that rewarded maintenance of a correct frequency and penalized frequency fluctuations was essential. The number of acts per minute is not necessarily a reliable parameter to tell an excellent performance (i.e., correct and uniform rate) from a sub-optimal one, such as correct but non-uniform rate with marked variations in frequency. With reference to Figure 5, we defined the sequence of acts 1, ⋯ , *n* and the corresponding Δ_*i*_: = *t*_*i*_−*t*_*i*−1_ as the difference between the time of act *i* and the time of the previous act *i*−1. The correct timing intervals are then defined [*min*_*freq*_, *max*_*freq*_] (40–60 ventilations per minute and 80–100 [+30] compressions per minute, where +30 represents the ventilations performed alternately by the virtual assistant). These ranges represent the reference values that the user must maintain and correspond to an interval [*min*_*timing*_, *max*_*timing*_] = [1/*max*_*freq*_, 1/*min*_*freq*_] between the minimum and maximum of the time interval allowed to perform a correct number of acts per minute. Therefore, the correctness value of the *i*th act is defined as follows:

(1) $$d_{i}:=\{{}_{max(|\Delta_{i}-min_{timing}|,|\Delta_{i}-max_{timing}|)\text{ ​​​​​​​​​​ }otherwise}^{0\;if\;\Delta_{i}\in [min_{timing},max_{timing}]}$$

With reference to Figure 5, every act falling within the correct ranges is rated as zero, while any variation outside the range (in red in the figure) increases the score in proportion to how much it deviates from the reference values. The first score is defined as the average of the ${\left\{d_{i}\right\}}_{i=1}^{n}$ [e.g., $score_{mean}=mean_{i=1}^{n}\left(d_{i}\right)$]. A null score represents a candidate who has always maintained an optimal frequency of acts while a higher score identifies any deviation from the correct execution. The second score is based on the standard deviation of the ${\left\{d_{i}\right\}}_{i=1}^{n}$ [e.g., $score_{std}=std_{i=1}^{n}\left(d_{i}\right)$]. This score characterizes the irregularity of the values and is, therefore, indicative of maintaining a non-homogeneous timing during the test.

### Ethical Approval

Users were pediatric/neonatology residents of the Azienda Ospedaliera Universitaria Pisana (AOUP) who consented to the acquisition, processing, and dissemination of data in anonymized form. The study was approved by the local Institutional Review Board for Ethic Issues. All analyzed data were anonymized and the entire analysis was blinded.

### Statistical Analysis

The study design is based on a stratified random sampling to control the nuisance factors. The strata are designed on the basis of a score extrapolated from a questionnaire of previous theoretical/clinical/practical experience. This score was used to create four levels of competence (0 no experience, 1: one of the three experiences, 2: two experiences, up to 3 for those who participated in all simulation, theory, and practice experiences), then used in the study design to divide the candidates of the two groups. The uniformity of the knowledge test 0 score distributions of the two groups' clinical experience was tested using a Kolmogorov–Smirnov (KS) two-sided test. A further indicator of uniformity is the amount of times a random sampling could have produced a better subdivision than the chosen design. This estimate was achieved by using a Monte Carlo method for probability estimation: 100,000 times the group of all candidates (associated with their respective knowledge test score 0) is randomly divided into two groups (27 and 21, respectively). This (artificial) subdivision represents a possible result of a random fully experimental design. Then, the Kolmogorov–Smirnov distance *D* between the two sets is calculated and compared with that obtained in the stratified random sampling. The knowledge test scores calculated before learning, at the end of learning and 28 days later, were evaluated by comparing the means, variances and distributions (KS test). The normality of the scores obtained was tested by Shapiro–Wilk test. Variances were compared by *F*-test for (independent) groups comparison and by Pitman–Morgan test of variance for paired sample for internal group comparisons. Under the assumptions of normality and homogeneity of variances, the independent *t*-test was used to compare means. In the absence of these hypotheses, the non-parametric (conservative) Wilcoxon signed-rank test and the Mann–Whitney *U*-test were used. Considering that the scores calculated in the knowledge tests 0, 1, and 2 are repeated measures of the same group and the frequent absence of the hypothesis of normality, the values are preliminary compared using a Friedman test. Post hoc pairwise analysis through the previously described paired tests are then applied to detect variations of the score. Bonferroni correction is presented to counter the problem of multiple post hoc analysis. The comparison between independent groups (i.e., DGBL vs. theory) pre-training, at 1 day and at 28 days is instead carried out with non-paired tests. To analyze the performance of the individual game sessions, the same tests were applied to learning score procedure, the response times of the questions, and the uniformity of the ventilation/compression timing. One-sided versions of the tests were applied to test the monotony of the scores. Statistical analysis was carried out using the software R [4.1.1] (27).

## Results

### Participant Characteristics and Stratified Random Sampling

Sixty-three pediatric/neonatology residents from the Azienda Ospedaliera Universitaria Pisana (AOUP) were recruited for the study, ranging from the first to the fifth specialty year with a high variability in previous training. The level of competence of each resident depends on the experience acquired before the start of the analysis (year of specialty, practice using a simulator, having attended theoretical training, and also real clinical practice with newborns). These nuisance variables (i.e., a variable that may alter the outcome of the study but is of limited interest in the chosen design) were of no interest to the study and had to be controlled to ensure homogeneity of the two groups using the stratified random sampling. By applying the Monte Carlo approach against the Kolmogorov–Smirnov distance calculated with the chosen design (d = 0.13), only 8% of the random subdivisions thus generated show a distance *D* < *d* = 0.13, confirming the validity of the design used.

**Figure 6:**
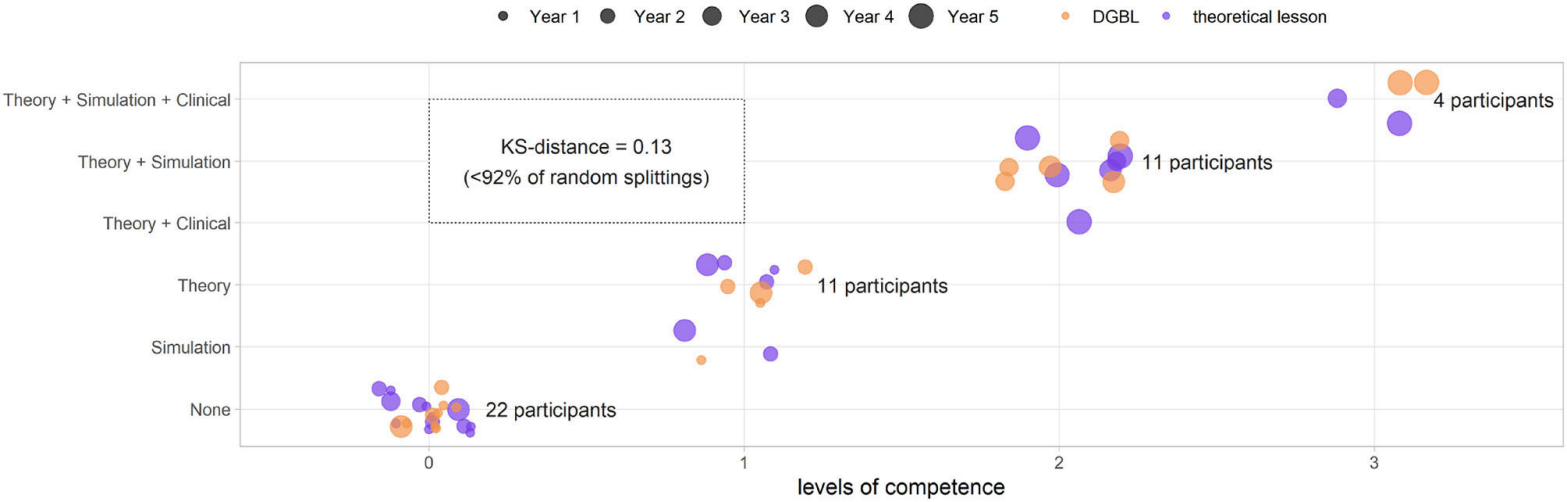
Group subdivision based on competence levels for the stratified random sampling (digital game based learning [DGBL] group in orange, theoretical teaching session group in purple). Using a Monte Carlo approach based on the knowledge test 0 score and the Kolmogorov–Smirnov distance, it can be shown that this subdivision is better than 92% of those artificially obtained through a fully random design.

Furthermore, the validity of the study design was tested also by comparing the knowledge test 0 and the check equipment scores between the two groups: no statistically significant differences were found (two-sided Mann–Whitney *U*-test *p* = 0.21≫0.05 and two-sided independent *t*-test *p* = 0.51≫0.05 for equipment score). Furthermore, the distributions of both values were also not dissimilar (two-sided Kolmogorov–Smirnov test, *p*≫0.5). The experiment design, and the corresponding subdivision of the population in strata, allowed to obtain a homogeneous level of past experience (as shown by the level of competence in Figure 6). The two groups were therefore considered uniform in the baseline scores (knowledge test 0) and homogeneously subdivided according to the confounding variables.

**Figure 7:**
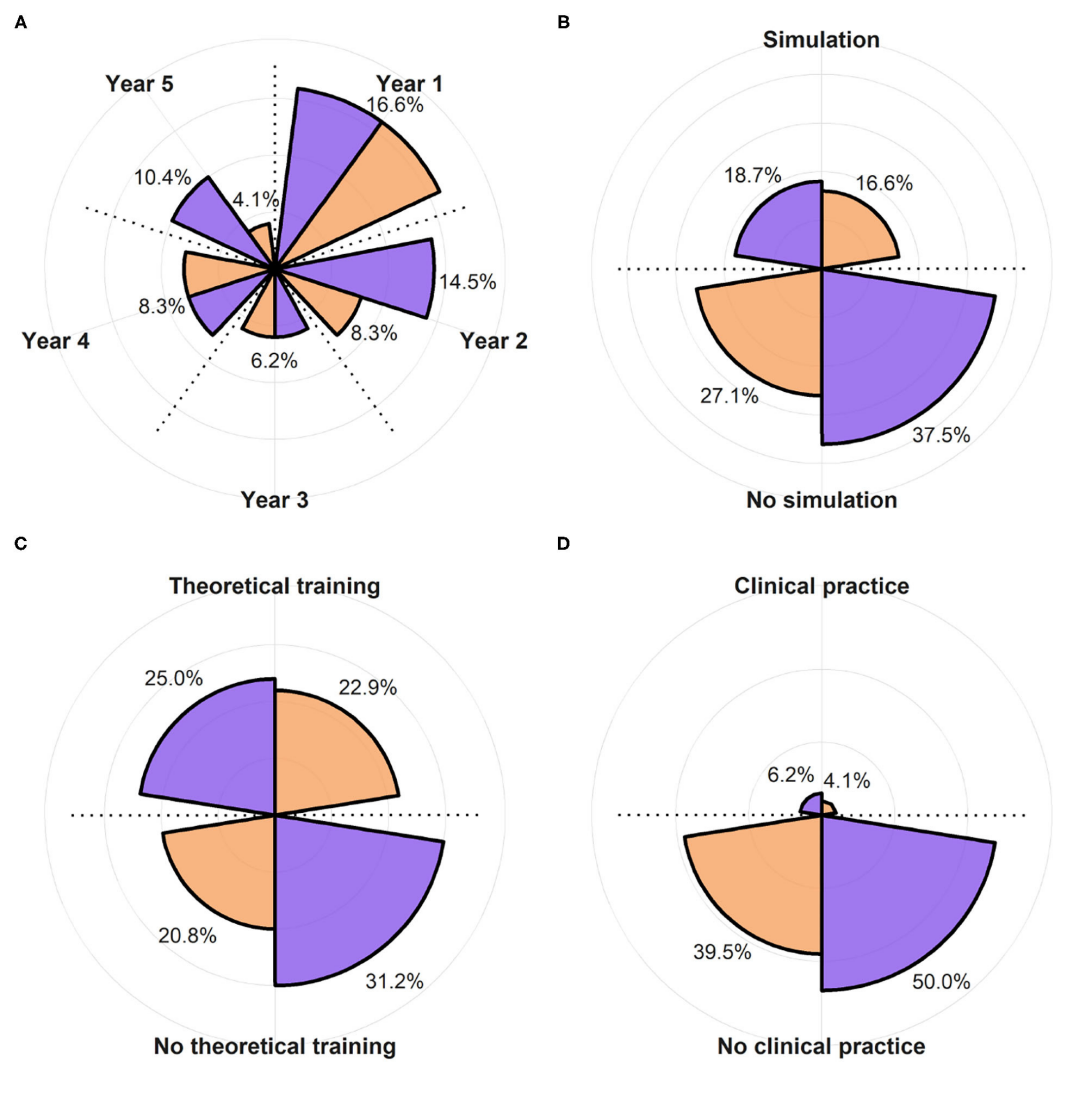
Subdivision of the population of the study between DGBL group (in orange) and classic theoretical teaching group (in purple). **(A)** Shows the year of specialty training (not one of the variables considered in the stratified random sampling) and is therefore characterized by a higher variability. **(B–D)** Show the percentage of the subjects that had used a newborn clinical simulator, underwent theoretical training in neonatal resuscitation, and practiced in neonatology, respectively.

The design led to two groups uniform in terms of previous experiences (Figure 7). Candidates who dropped out for personal reasons, or those failed to meet learning and testing sessions deadlines, were excluded from the study: of a total of 15, the majority affected DGBL group, yielding 27 candidates for the classic learning group and 21 for DGBL group. In the breakdown of the study sample by specialty year, 56% of the residents clustered around first and second year (Figure 7A), only 35.3% of the trainees had practiced at the simulator before this study (Figure 7B), whereas 47.9% had already received theoretical training in neonatal resuscitation (Figure 7C). User characteristics that could significantly impact results (e.g., neonatal clinical experience, as shown in Figure 7D) were uncommon in this cohort (only 10.3% of candidates); this setting required a proper design in order to prevent concentrating the few candidates with any particular characteristic in only one of the two groups. Consequently, the reference sample can be described as having a dominant component of students of the first years, mostly with no previous experience (45.8%). The older residents were the ones with greater medical experience (clinical/simulation/theoretical), with all fifth-years students having received at least one theoretical teaching session and one practical tutorial at the simulator.

### Comparison Between DGBL and Classic Learning

#### Knowledge Retention

**Figure 8:**
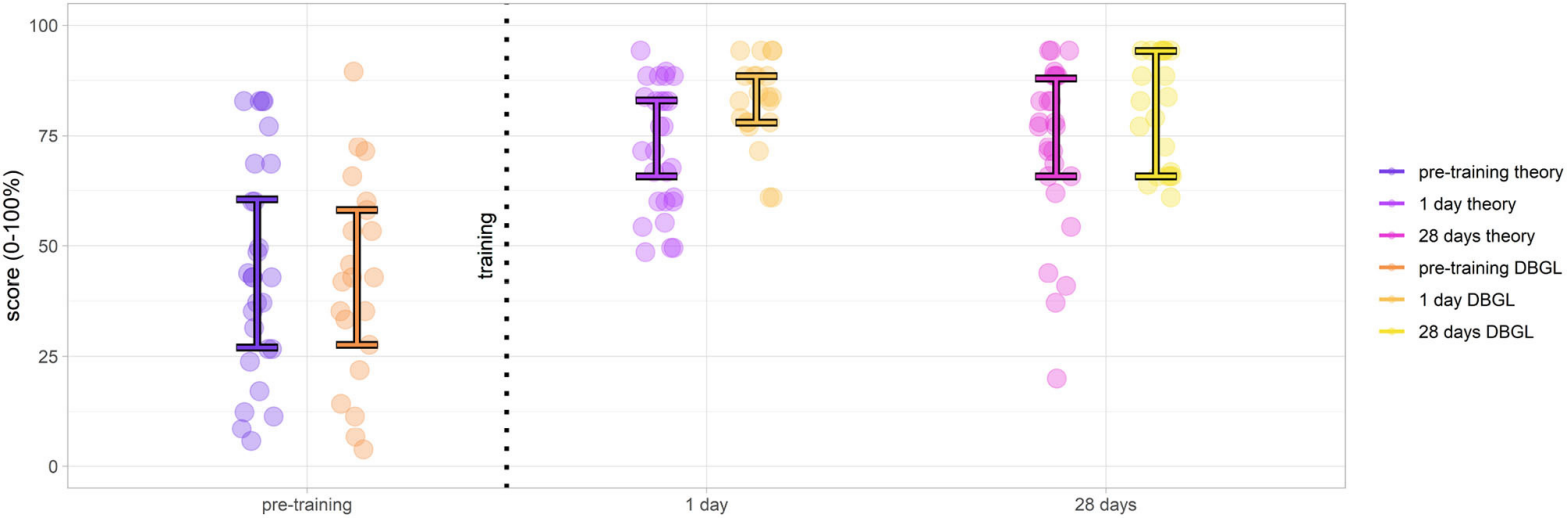
Results of knowledge tests evaluated pre-training, 1 day post-training, and at 28 days follow-up (score medians and middle 50% interquartile ([0.25, 0.75]); theoretical teaching session group scores in purple, digital game based learning (DGBL) method group scores in orange). Although pre-training groups are comparable, post-training scores demonstrate the effectiveness of both methodologies and DGBL, in particular.

The first analysis was based on the scores obtained in the knowledge tests 0, 1, 2 (respectively, pre-training, 1 day post-test, and 28 days later follow-up). None of the observed test score distributions could be assumed to be normal except pre-training scores (Shapiro–Wilk test, α = 0.05) as shown in Figure 8 (purple for classic learning and orange for DGBL approach).

After a preliminary Friedman test (α = 0.05) that found differences in scores between the knowledge tests 0, 1, 2 for both the DGBL (*p* ≪ 0.001) and the theoretical training (*p* ≪ 0.001), we moved on to the post-hoc pairwise analysis. The effectiveness of the theoretical teaching session was proved by an increase in pre-training and post-training tested scores at 1 day, with an increase in median scores from 42.8 to 71.4% (paired one-sided Wilcoxon signed-rank test, *p*≪ = 0.001). An even greater increase in scores was found for DGBL training, with median scores ranging from 42.8% pre-training to 83.8% post-training (paired one-sided Wilcoxon signed-rank test, *p*≪0.001). There was no statistically significant reduction in scores following the 28-day wait (α = 0.05). Even considering a conservative Bonferroni correction factor (*m* = 2) to control the family-wise error rate, the reported results have much lower p-values than the corrected $\tilde{\alpha }=\alpha /m=0.025$. The initial pre-training scores could be considered coincident both as medians (two-sided Mann–Whitney *U*-test, *p* = 0.21≫α = 0.05) and as distributions (two-sided Kolmogorov–Smirnov test, *p* = 0.97≫α = 0.05). This allowed to compare the score increases for the two methodologies. Therefore, considering the post-/pre-training score differences, DGBL method was statistically not inferior to the classic teaching session (one-sided Mann–Whitney *U*-test, *p* = 0.005). As represented graphically in Figure 8 (28 days), score variance decreased between pre-training and post-training (1 day) for both methodologies (one-sided paired Pitman–Morgan test, *p*≪α = 0.001). There was no statistically significant variance increase 28 days post-learning for DGBL group (*p* = 0.07 > α = 0.05), while variance increased significantly for the classic methodology group (*p* = 0.02 < α = 0.05). Furthermore, the variance at 28 days for the classic learning group was greater than that of DGBL group, with values more distributed over the score range (one-sided *F*-test, *p* = 0.03 < α = 0.05). The variance of the analyzed scores makes it possible to distinguish between a population with a homogeneous knowledge (low variance) compared to one with marked differences between the scores of the individuals (high variance). For this reason, we want to investigate whether following learning there is a simple increase in scores, which is an indication of an effective transmission of knowledge, or even a consequent reduction in the variance of scores, that is representative of uniformity of skills following learning (e.g., we were able to teach them what we wanted to teach them).

#### Equipment Game

**Figure 9:**
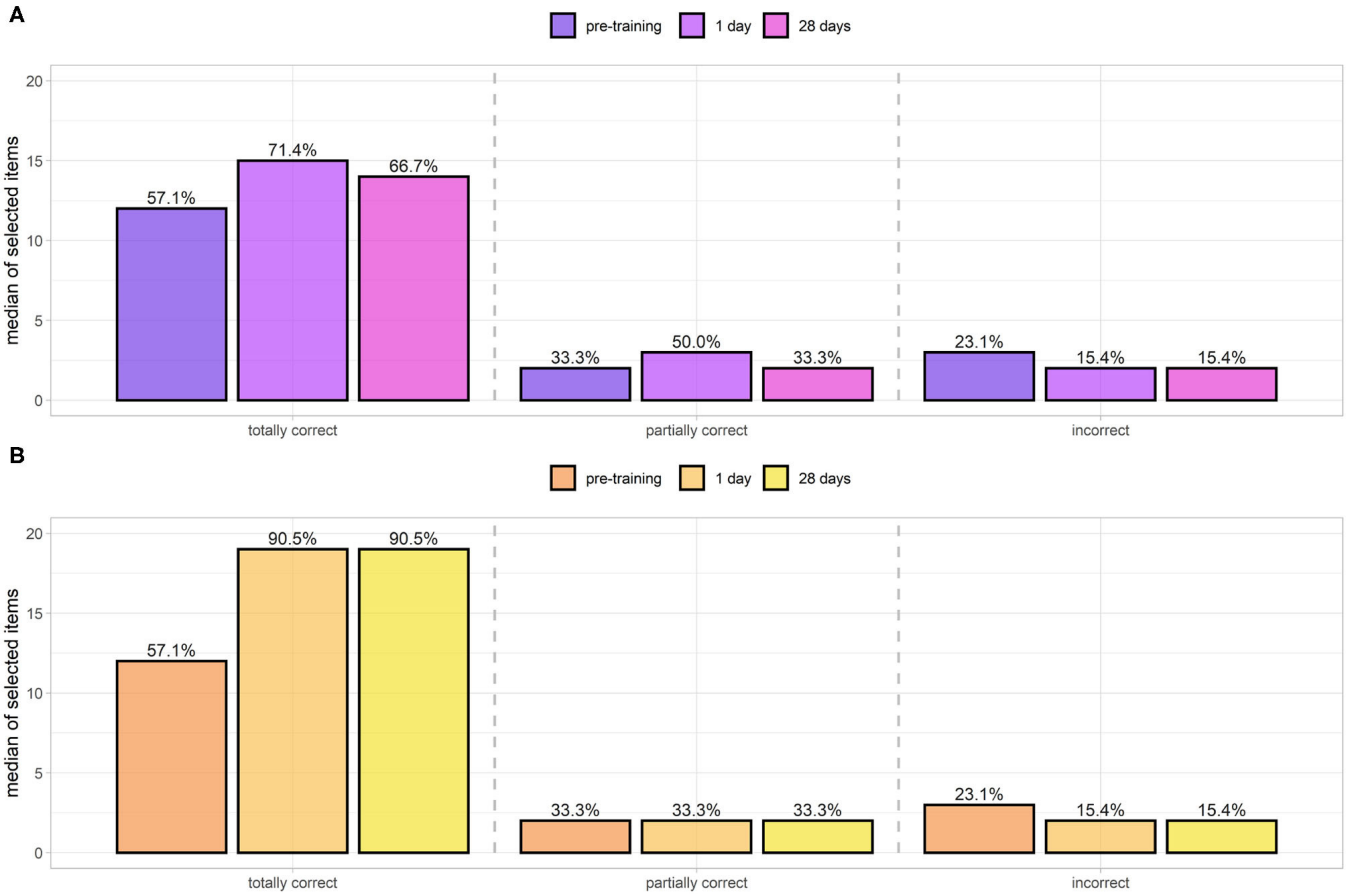
Equipment scores of totally correct/partially correct and incorrect items selected by the control group (standard teaching session, **A**) and digital game based learning (DGBL) group **(B)**. Initial equipment scores for the two methodologies are not statistically different. DGBL methodology leads to a greater increase in correct item selection. It also reduces selection of incorrect/partially correct items, whereas after theoretical teaching no reduction is observed.

Equipment scores were divided into three categories: totally correct, partially correct, and incorrect. Learning was considered to be effective if users selected a greater number of correct options and fewer of the incorrect/partially correct ones. The scores evaluated at steps 0, 1, 2 (respectively, pre-training, 1 day post-training, 28 days follow-up) of classic learning (in purple, Figure 9A) and DGBL methodology (in orange, Figure 9B) are shown in Figure 9. All the score distributions were non-normal, except scores for the correct tools at the 0/pre-training evaluation (Shapiro–Wilk, α = 0.05). A preliminary Friedman test (α = 0.05) is performed to detect if there is a difference among the three assessments (knowledge test 0, 1, 2) for both DGB/classic learning and for the totally/partially correct and incorrect items. A statistical significance of the learning effect is only found for the totally correct items (classical theoretical learning, *p* = 0.02) and for both totally correct (*p*≪0.001) and incorrect (*p* = 0.007) items score for the DGBL training. Classic learning (Figure 9A) was effective in achieving memorization of totally correct objects (57.1 to 71.4%, paired one-sided Wilcoxon signed-rank test con *p*≪0.001). No other statistically significant improvement (α = 0.05) was noted in any of the other scores, either in relation to the 1 or 28-day assessment. Conversely, there was an increase in the partially correct objects chosen in Test 1 (33.3 to 50.0%, *p* = 0.01). The DGBL methodology proved more effective (Figure 9B), with not only a statistically significant improvement in pre-training/ 1-day scores for correct items (57.1 to 90.5%, paired one-sided Wilcoxon signed-rank test con *p*≪0.001), but also with a moderate a reduction of incorrect items (23.1 to 15.4%, *p* = 0.03), which is not statistically relevant for the classical learning method. The initial scores for the correct objects coincide for the two groups for both the median (57.1%, paired two-sided Wilcoxon signed-rank test con *p* = 0.72≫0.05) and the mean values (59.2% theoretical teaching session, 56.7% DGBL, two-sided independent *t*-test, *p* = 0.51≫α = 0.05). As the coinciding baselines allow an analysis of the pre-/post-training differences of the two groups, the DGBL methodology led to a significantly greater improvement than the classic learning one (one-sided Mann–Whitney *U*-test, *p* = 0.009 < α = 0.05). We did not carry out the same analysis for partially correct and incorrect objects, as uniformity between the two strategies cannot be assumed at the α = 0.05 level. In this analysis, considering a Bonferroni correction factor (*m* = 2) did not change the result of the effect of training on score of correct items. However, the effect of the reduction of incorrect items for the DGLB group can no longer be considered statistically significant.

#### Item Choice in Equipment Check

**Figure 10:**
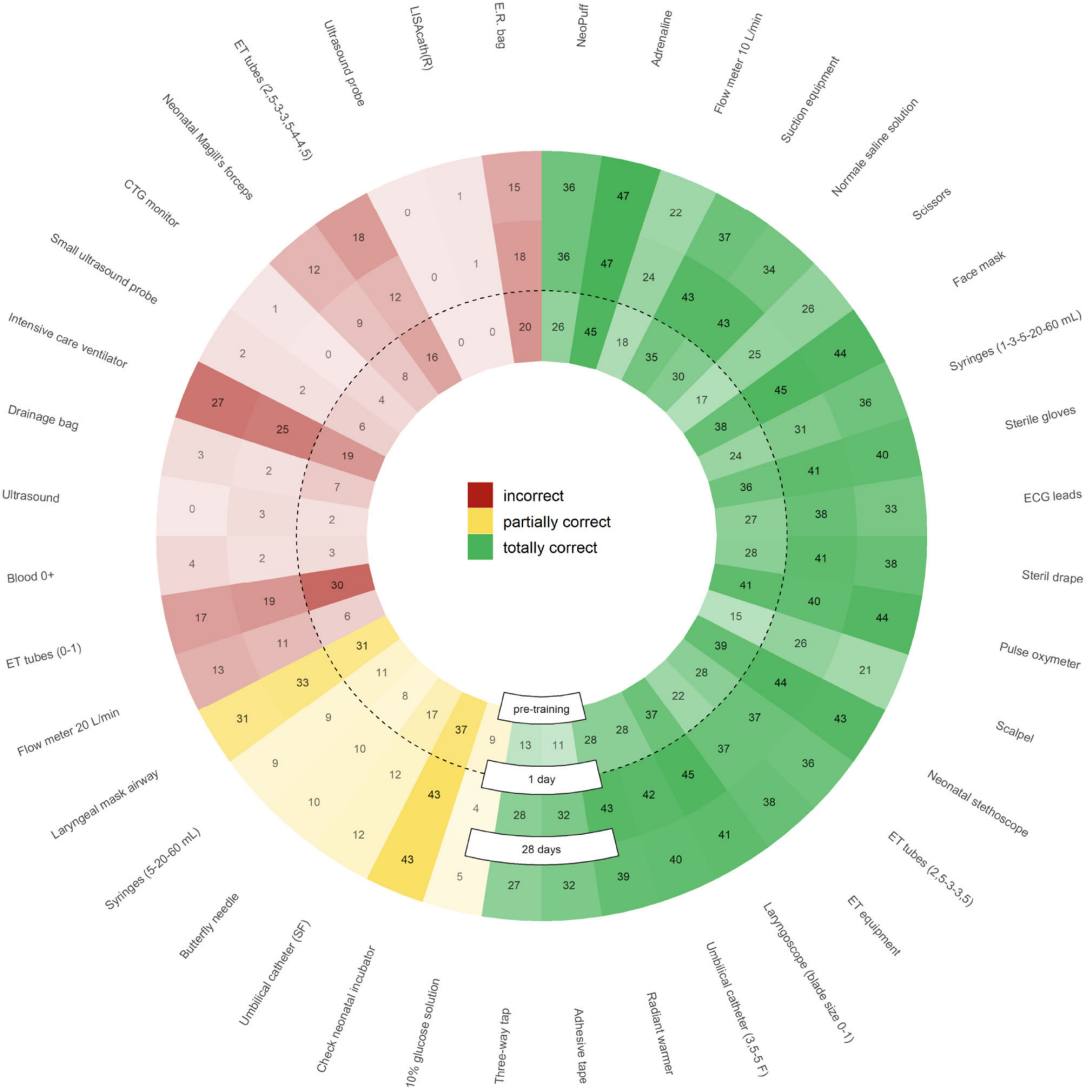
Number of items selected by users (regardless of learning mode) during the knowledge tests, divided by color into incorrect (red), partially correct (yellow), and totally correct (green). Greater color opacity indicates a greater number of selected items. Selected elements numbers are subdivided as pre-training (inner circle), 1-day post-training (middle circle), and at 28 days follow-up (outer circle). Color opacity shifts highlight the items for which learning has proved particularly effective (e.g., ECG leads that go from 27 pre-training to 38/33 post-training or ET tubes (size 2.5, 3, 3.5) that are reduced from 30 to 19/17), whereas uniformity of color opacity across the concentric circles show the items for which learning has proved ineffective (e.g., intensive care ventilator, laryngeal mask airway, or check neonatal incubator).

With regard to the total number of times that each tool was selected during the knowledge tests (regardless of the learning mode), training methodology can be improved. Indeed, Figure 10 highlights the elements for which the methodology worked well (increasing values for totally correct from the center outwards, and decreasing for partially correct/incorrect ones) and those for which it does not (stable scores among the sessions). For almost all totally correct options, learning proved effective with both methodologies; however, some options were too obviously correct, e.g., adrenaline administration (45–47/47) or pulse oximeter use (41–40/44). The best training effect was seen on discouraging the selection of endotracheal tubes (ET) (0, 1) (30–19/17). For other incorrect items (intensive care ventilator, E.R. Bag, E.T. tube size) and partially correct ones [laryngeal mask airway (31–33/33), check neonatal incubator (37–43/43), E.R. bag (20–18/15), and intensive care ventilator (19–25/27)], the learning was not effective enough, as users continued to rate them as necessary despite training indicating otherwise. We are planning the implementation of software changes, which will allow to investigate communication effectiveness for these learning objectives. It should be emphasized that some incorrect tools [ultrasound machine, ultrasound probe, LISAcath(R)] proved poor distractors, as users hardly ever selected them. Therefore, future versions of the game will not include those items.

### DGBL Game Performance

#### Knowledge Retention

**Figure 11:**
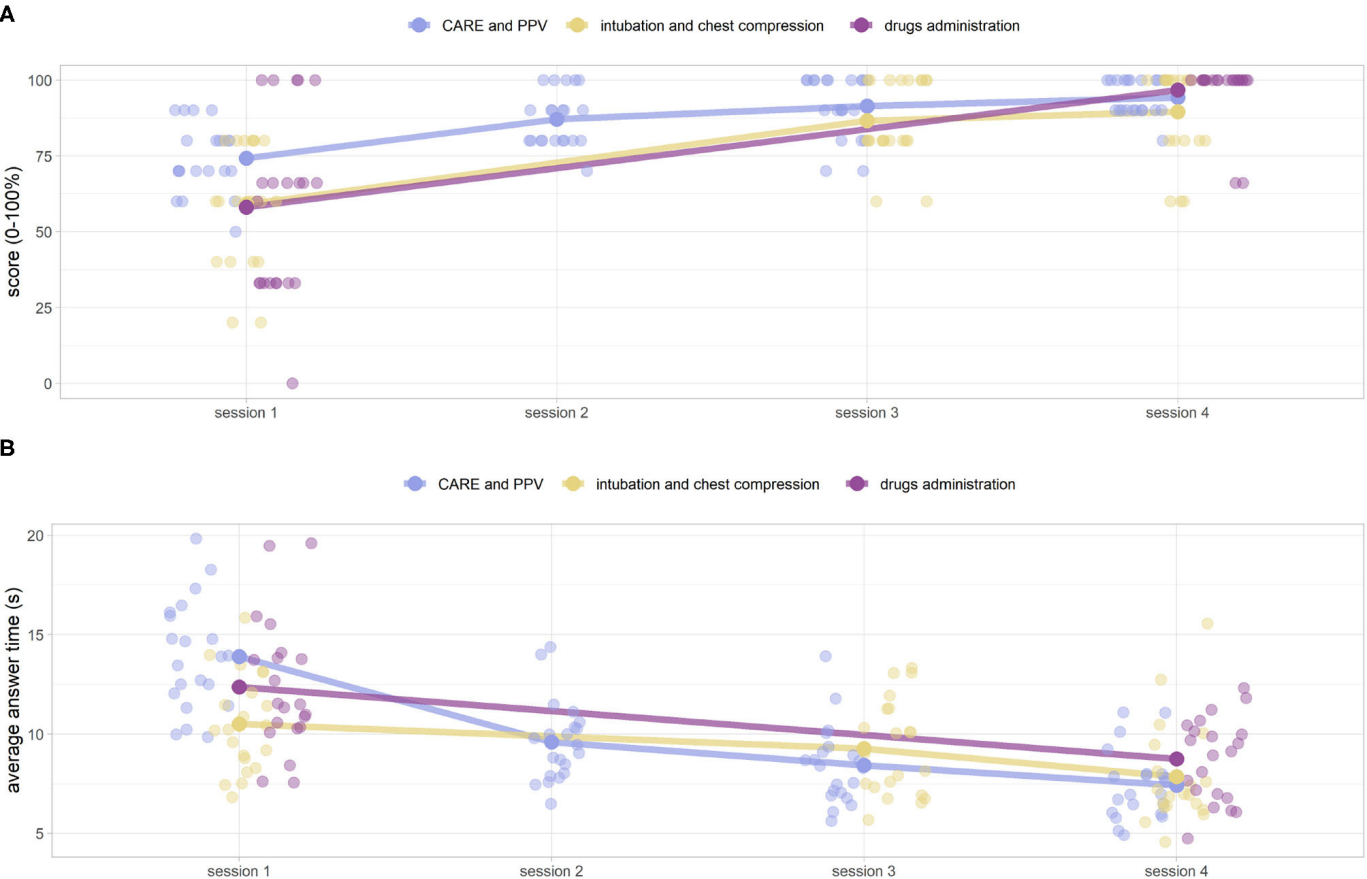
**(A)** DIgital Application in Newborn Assessment (DIANA) game scores, and corresponding median values, over the four sessions. The scores are subdivided into three categories (CARE and PPV, intubation and chest compressions, drugs administration) for ease of analysis. **(B)** Corresponding average answer times (in seconds).

Figure 11A shows the scores and the respective averages of the game sessions (e.g., the number of correct answers) by category and session number (1, 2, 3, 4). The medians response times (seconds) for the entire corresponding series of questions are shown in Figure 11B. After three preliminary Friedman tests for scoring (CARE and PPV, intubation and chest compression, and drug administration) and three more for answer times (α = 0.05), all identifying a statistical difference, we moved on to post hoc pairwise analysis. Both panels show a strong monotonicity in the functions, with increasing scores (~65.3 to 96.7) and decreasing times (~11.9 to 7.7 s) as the sessions progress (one sided paired Wilcoxon signed-rank test, α = 0.05), except for equipment CARE and PPV/intubation and chest compression scores between session 3 and session 4 (α = 0.05), which did not show a statistically significant increase (knowledge plateau). After a Bonferroni correction by a factor (*m* = 5) for the CARE and PPV and a factor 3 for intubation and chest compression, the same results remained valid at a level of $\tilde{\alpha }=\alpha /m=0.01$ except for the CARE and PPV scores between session 2 and 3, whose increase was no longer statistically significant. Test values at sessions 1 and 4 highly correlate (ρ = 0.84, Pearson test for linear correlation *p*≪0.001) with knowledge test scores 0 and 4. The game scores are therefore predictive of success in the following knowledge test.

#### Equipment Game

**Figure 12:**
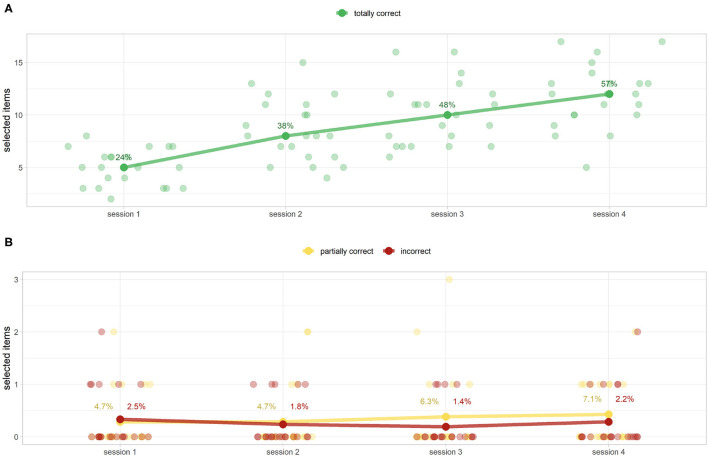
DIgital Application in Newborn Assessment (DIANA) game equipment scores (as selected items and their percentage of the median of total items) for totally correct **(A)** and partially correct/incorrect items **(B)**. **(A)** Shows a monotonous increase of correctly chosen elements (from 24 to 56%). **(B)** Shows extremely low values of partially correct/incorrect scores. This finding reinforces the idea that visual memory plays a pivotal role in memorization. **(B)** Shows the percentage of the mean values because the corresponding medians are all equal to 0.

As anticipated by knowledge tests (Figure 8), the sessions improve users' ability to choose the correct objects. Indeed, the number of totally correct objects selected (Figure 12A) and partially correct/incorrect ones (Figure 12B), respectively, increased and decreased after each session. Specifically, since all the scores are not normal (Shapiro–Wilk test at the alpha level = 0.05), we proceeded to test the monotony of the score with a non-parametric test (preliminary Friedman test at a level α = 0.05 that revealed a statistical difference between the totally correct scores, and *post-hoc* one-sided paired Wilcoxon signed-rank test, respectively, *p*≪0.001, *p* = 0.01, *p* = 0.003 between sessions 1–2, 2–3, and 3–4). Unlike procedure memorization highlighted by the scores (Figure 11), there is still a statistically significant improvement for this game between sessions 3 and 4 (*p* = 0.003). A Bonferroni correction factor *m* = 5, the number of pairwise analysis carried out, can be applied ($\tilde{\alpha }=\alpha /m=0.01$). Despite the correction, the results presented remain unchanged. Furthermore, candidates made fewer mistakes when the tool name was paired with its picture (medians of 0 for partially correct items and incorrect ones vs. 33.3 and 15.4% for the same students during the knowledge test), as shown in Figures 12B, 9B, respectively.

#### Ventilation and Compression Game

**Figure 13:**
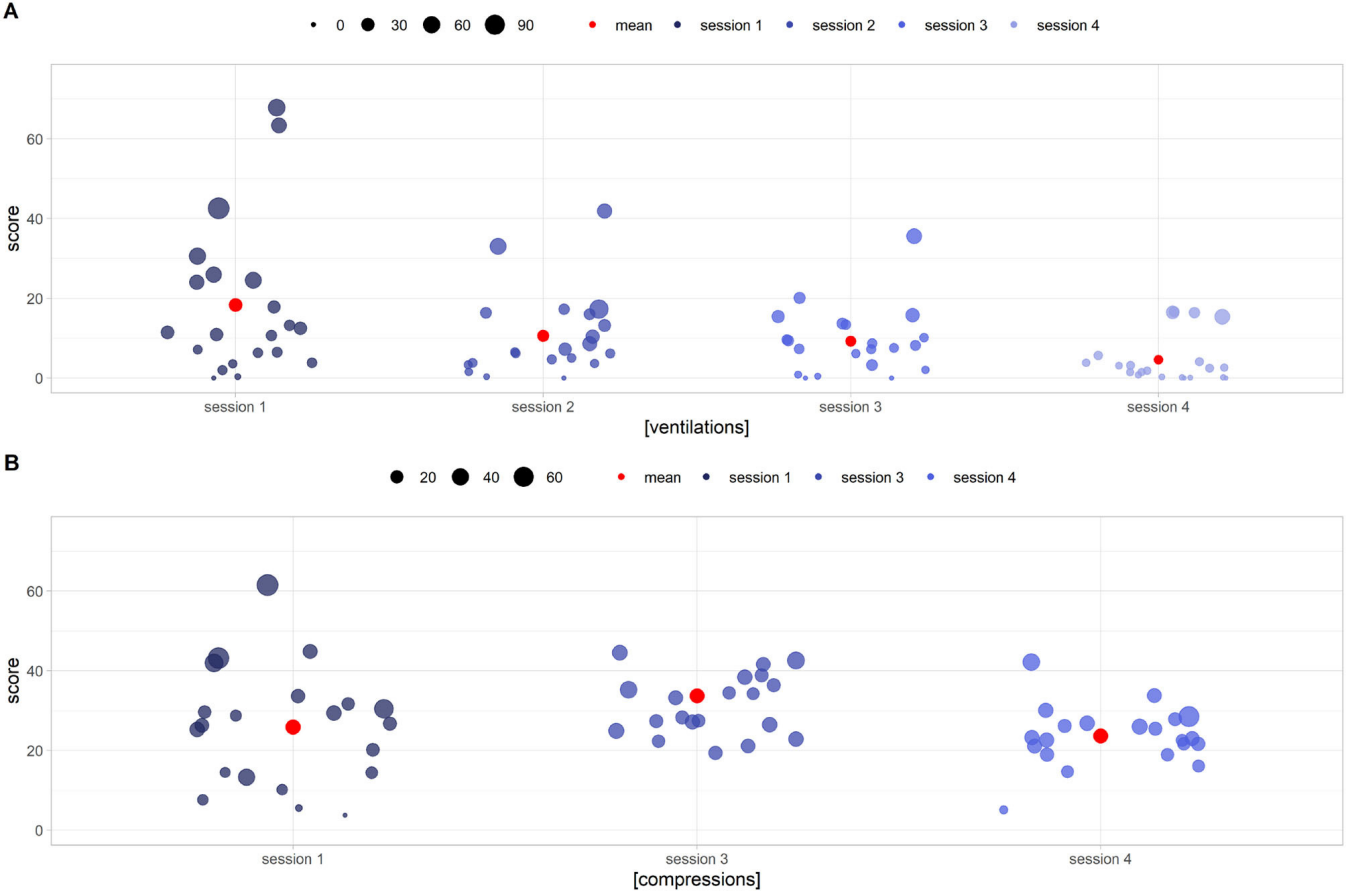
DIgital Application in Newborn Assessment (DIANA) game scores (y axis) and standard deviation (circle size) for ventilation **(A)** and chest compression **(B)** execution. Low y values imply an execution frequency closer to the correct one, while short circle radii identify smoother acts. **(A)** Shows performance improvement in terms of both correct frequency (decreasing score to 0) and smoothness of execution (small circles radii). **(B)** Shows a less noticeable improvement in performance, with users still unable to execute compressions correctly after the fourth session.

Figure 13 shows the deviation from the correct ventilation (40–60 breaths per minute = intervals between 100 and 150 hundredths of a second) and compression ranges (80–100 breaths per minute = considering 30 alternating breaths per minute, intervals between acts of 46–54 hundredths of a second). The score (Y axis) represents the precision of execution in terms of number of acts, a score of 0 representing a frequency kept within the range. Circle size is the standard deviation (STD) of the uniformity score *d*. Smaller circles represent greater execution uniformity. Ventilation frequency was not as effectively learned as compression rate, despite the apparent similarity of the games aiming to teach them (Figures 13A,B, respectively). From a statistical point of view, the values of the scores and the STDs of the rates all follow non-normal distributions (Shapiro–Wilk test, *p* < 0.001 for scores and *p* < 0.01 for STDs). Regarding the ventilations there is an improvement of both parameters. Score values are decreasing with monotony (one tailed Wilcoxon signed-rank test *p* < 0.01). With regard to STDs, on the other hand, there is a statistically significant reduction between the first/second session (*p* = 0.03) and the third/fourth session (*p* = 0.001) but not between the second/third. The same tests, applied to compressions, were all statistically inconclusive.

#### Satisfaction Questionnaires

Of the data collected from DGBL user satisfaction questionnaires (20/21), we evaluated perceived utility and enjoyment of the procedure (both using a five-level Likert scale). Ratings were generally positive in terms of perceived utility [40% (5/5) and 60% (4/5)] and procedure agreeableness [40% (5/5) and 60% (4/5)]. Suggestions mainly concerned the need to increase available equipment game time, perceived as too short. Positive feedback was given on spreading the game sessions over different days.

## Discussion

This study successfully applied a DGBL-based approach to neonatal resuscitation teaching through the use of a newly developed software (DIANA). DIANA game focused on the entire neonatal resuscitation algorithm (including equipment check, neonatal care, drug administration, assisted ventilation, and chest compressions). The study was aimed at pediatric/neonatology residents [a learners' category considered in few studies as in the mixed study group by (12)], while the majority of previous findings in this field have focused on undergraduate medical students (9, 19, 20), healthcare professionals (11, 12, 21), and experienced neonatal providers (17, 21). This study's sample size is similar to other DGBL studies in the medical/neonatal field (9, 10, 17, 28, 29). Learner allocation (Stratified random sampling) to two experience-based groups (year of specialty training, previous theoretical teaching session, simulation experience, clinical practice experience) proved effective in obtaining homogeneous baseline scores (Section 3.1). Furthermore, the subdivision obtained was better than 92% of those eventually obtained by applying a completely random method. This study is among the few that (1) fully exploit the ability of a game to extract user data (e.g., ventilation/compression game scores, response time, etc.), (2) define a treatment group and an independent control group through a baseline score (pre-test), and (3) evaluate two follow-ups (short- and long-term knowledge retention). In addition, compared with the majority of published studies, which tested learner months apart (9, 10, 17), we preferred to keep the testing interval shorter [and yet longer than 2 weeks, in line with best practice in assessing DGBL learning (30)]; as the studied cohort was recruited among pediatric/neonatology residents, specialty training would invariably continue to provide reinforcement of the skills assessed. It should be noted that 28 days are considered a sufficient timeframe to evaluate memorization of a procedure in the medium to long term (25).

The DGBL methodology proved to be useful and appreciated by users to teach both neonatal resuscitation algorithm and ventilation execution. Furthermore, it proved to be even more effective than the classic frontal teaching session for both short-term procedure memorization and equipment game score. In particular, the scores related to short-term knowledge retention proved to be higher than those obtained by the theoretical frontal teaching session, in line with the existing literature (10, 19, 20). Also in line with the limited number of studies with a follow-up at more than 28 days (9, 10, 17), long-term knowledge retention for DGBL group was as good as the control group one. Furthermore, candidates who had received classic training demonstrated a regression to lower scores, unlike DGBL methodology learners. DGBL methodology was particularly effective in the learning of clinical equipment checking. Although the classic theory teaching session led to a statistically significant, but moderate increase in the number of correct objects chosen (57.1–71.4%), DGBL-based approach led to a much greater improvement (57.1–90.5%). Furthermore, while the classic teaching session had almost no effect on changing the scores for partially correct/incorrect items (33.1–50.0 and 23.1–15.4%), DIANA game reduced or leave unchanged scores for both partially correct (33.1–33.1%) and incorrect tools (23.1–15.4%). This score discrepancy is likely due to the difference in the way the learning objective is conveyed: DGBL approach breaks learning into sub-games (one of which is the explicit teaching of which tools should be used), while during a theory teaching session the tools are named progressively at the time of their use. Overall, the DGBL methodology subdivision of learning into multiple sessions was confirmed to be effective for the learning of neonatal resuscitation in line with further previous simulation-based studies (28, 29), especially for the maintenance of the acquired competencies (31). The information collected in the DGBL sessions allows performance analysis (learning curve) related to flowchart learning, response times, equipment check, and timing of assisted ventilations/chest compressions. Procedure learning was effectively achieved, in line with the existing literature (9, 17, 21): the first three sessions showed significant improvements in learning, while the fourth highlighted a learning plateau. Of note, there was a constant improvement in response times along the four sessions, with a total reduction of more than 30% of the initial one. Similarly, there was a steady improvement in the correct equipment check score (from 24 to 57%). In the assisted ventilation game, DGBL methodology proved to be effective, as residents responded to the feedback from the game and learned to keep the correct rate independently. However, in the chest compressions game, similar in execution to the assisted ventilation one, we did not observe the same effectiveness; candidates did not improve in either the frequency (remaining outside the required clinical range) or the regularity of compressions. This pattern persisted across all four sessions. The discrepancy between these two results could be induced by the differences between the two games. Indeed, during the compression game, the user must interact with the virtual assistant which performs ventilation. To complete the task before next the ventilation, users tend to perform excessively clustered and irregular compressions. To facilitate the reading of the discussion presented above, the results and consequences of the study are shown in Table 2.

**Table 2:** Summary of study results.

**Comparison**	**Results**
Primary endpoint	The DGBL methodology proved to be even more effective than the classic frontal teaching session for both short-term procedure memorization and equipment game score. Long-term knowledge retention for the DGBL group was as good as that of the control group (classical training).
Secondary endpoints	The answer time (seconds) decreased after each session. The score of the games increased for the first three sessions and then reached a plateau.
	The sessions improved users' ability to choose the correct equipment.
	There was a statistically significant improvement in the execution (rate) of ventilation after each session.
	There was no statistically significant improvement in the execution (rate) of chest compression
	Ratings were positive in terms of perceived utility and procedure agreeableness.

The administration of a user satisfaction questionnaire confirmed a greater appreciation for DGBL as a training methodology than the classic frontal theory teaching session, in line with the existing literature (13, 19, 20). DGBL methodology usability is crucial for future developments, as learners positively disposed to digital tools tend to respond more effectively (26). Based on the satisfaction questionnaire results, appreciation was lower for the check equipment game compared to the others, despite its effectiveness on improving user scoring.

One of the limitations of this study is the inability of digital software to teach the execution of technical skills. Particularly for complex tasks (also to be combined with another operator), such as chest compressions, this methodology proved ineffective: users' acts remained too frequent, inappropriately clustered and not coordinated with the virtual assistant. Furthermore, the knowledge test does not guarantee that users will apply those skills effectively in a clinical context. Future versions of this software will be developed from the analysis presented in this study and the suggestions collected through the satisfaction questionnaire. Specifically, we aim to reduce the number of sessions to 3 (learning plateau detected at the fourth one), allow no time limitation for the equipment check game, and exclude from the tool list the obviously incorrect options (poor distractors). To overcome the limitation of learning technical skills in DGBL methodology, future developments may require integration with a physical support structure to allow the candidate to practice clinical tasks. To improve the application of these training methodologies, we are developing the online implementation of DIANA (both in Italian and English) to allow the autonomous use of DIANA in further medical realities, as a free tool for training and re-training. Because of the online platform, we are already extending the same analysis on a wider population. In this way, we can use our findings (on both population characteristics and expected scores) to estimate the required sample size to improve future studies. The future development of a hardware device for the execution of practical skills will also allow to overcome a known limitation in simulation field (i.e., by lack of a report on the technical performance of the user with high-fidelity mannequins). A high-fidelity simulator could offer a report on the correct execution of the flowchart based on human external observation of simulation. However, with a hardware device designed to record the events performed, both in terms of decision-making and practical performance, it will be possible to conduct a more detailed and precise study of the effectiveness of these two training methodologies.

We will also seek to modify the software with/without hardware integration to widen the potential user base, including other clinical specialties and varying levels of experience. In particular, we aim to extend this learning tool to users less accustomed to digital technology to further assess the impact of user mindset on the effectiveness of DGBL methodology (26). Moreover, as DGBL is unlikely to be adopted as a stand-alone teaching method (11) [especially in higher education (14)], future research may involve using the two methods in sequence, e.g., reinforcing the classic theory teaching session by DGBL, or a simulator-based introduction to a classic teaching session. This blended approach has been already validated for simulations outside neonatology (32). Considering the positive feedback obtained by remotely testing DGBL in other healthcare education contexts (17), deployment of DGBL to support healthcare education in low-income countries could represent another future development in the use of this learning technology.

## Conclusion

In this study, DGBL methodology for pediatric/neonatology resident training proved to be superior to theoretical teaching session (led by a neonatal expert trainer) on short- and long-term knowledge retention of memorization of the correct equipment to assemble. In addition, DGBL proved to be at least as effective as the teaching lesson for memorization and retention of neonatal resuscitation algorithm. DIANA game allows individual user session analysis, with an improvement in “session-after-session” scores and a reduction in decision-making times. We propose that DGBL could be a valuable addition to classic learning methodology for all medical procedures involving a procedural algorithm.

## Data Availability Statement

The raw data supporting the conclusions of this article will be made available by the authors, without undue reservation.

## Ethics Statement

The studies involving human participants were reviewed and approved by Dipartimento Materno-Infantile, Azienda Ospedaliero Universitaria Pisana. The patients/participants provided their written informed consent to participate in this study.

## Author Contributions

SB fully implemented the software DIANA, collected the data, drafted the initial manuscript, reviewed, and revised the manuscript. GD designed the study and the data collection tools, analyzed the data, carried out statistical analysis, drafted the initial manuscript, reviewed, and revised the manuscript. MD and FL collected the data, were involved in the classic training sessions, and revised the manuscript. RTS, MC, and AC wrote the knowledge test, taught during the classic training sessions, integrated the analysis with the corresponding medical discussion, and reviewed and revised the manuscript. NF reviewed, critically analyzed, and revised the manuscript including revision of English language. All authors contributed to the article and approved the submitted version.

## Conflict of Interest

The authors declare that the research was conducted in the absence of any commercial or financial relationships that could be construed as a potential conflict of interest.

## Publisher's Note

All claims expressed in this article are solely those of the authors and do not necessarily represent those of their affiliated organizations, or those of the publisher, the editors and the reviewers. Any product that may be evaluated in this article, or claim that may be made by its manufacturer, is not guaranteed or endorsed by the publisher.
