# Supplementary material for: Improving Pediatric/Neonatology Residents' Newborn Resuscitation Skills With a Digital Serious Game: DIANA
Source: Front Pediatr. 2022 Apr 1;10:842302. doi: 10.3389/fped.2022.842302 (PMC9010974; doi:10.3389/fped.2022.842302)
Supplement: Supplementary file 1 [file Data_Sheet_1.PDF]

Supplementary Material

1 SUPPLEMENTARY TABLE

EQUIPMENT IDENTIFICATION	OPTIONS	CLASSIFICATION
NEOPUFF		Totally correct
FLOW METER	20 L/Min O^2 = 21%<br>10 L/Min O^2 = 30%<br>20 L/Min O^2 = 30%<br>10 L/Min O^2 = 21%	Incorrect<br>Incorrect<br>Incorrect<br>Totally correct
NORMAL SALINE		Totally correct
ENDOTRACHEAL TUBE	Size 0-1<br>Size 2,5-3-3,5<br>Size 2,5-3-3,5-4-4,5<br>Size 1,5-2-2,5	Incorrect<br>Totally correct<br>Incorrect<br>Incorrect
SUCTION EQUIPMENT		Totally correct
ADRENALINE	1:10.000 ET SOMMINISTRATION 0,3 mL/Kg<br>1:10.000 ET SOMMINISTRATION 1 mL/Kg<br>1:100.000 ET SOMMINISTRATION 0,3 mL/Kg<br>1:100.000 ET SOMMINISTRATION 1 mL/Kg	Incorrect<br>Totally correct<br>Incorrect<br>Incorrect
SCISSORS		Totally correct
FACE MASKS		Totally correct
STERILE GLOVES		Totally correct
SYRINGE	-1/3/5/20/60 mL<br>-3/5/60 mL<br>-5/10/20 mL<br>-10/20 mL	Totally correct<br>Partially correct<br>Partially correct<br>Partially correct
ECG LEADS		Totally correct
UMBILICAL CATHETER	-5 F<br>-3,5-5 F	Partially correct<br>Totally correct
PULSE OXIMETER		Totally correct
STERIL DRAPE		Totally correct
SCALPEL		Totally correct
NEONATAL STETHOSCOPE		Totally correct
LARYNGOSCOPE BLADE	- SIZE (0-1)<br>- SIZE (3-3,5)	Totally correct<br>Incorrect
RADIANT WARMER		Totally correct
ADHESIVE TAPE		Totally correct
THREE-WAY TAP		Totally correct

Table S1. Totally correct equipment items defined in DIANA game.

EQUIPMENT IDENTIFICATION	OPTIONS	CLASSIFICATION
10% GLUCOSE SOLUTION		Partially correct
CHECK NEONATAL INTUBATION		Partially correct
BUTTERFLY NEEDLE		Partially correct
NEONATAL MAGILL'S FORCEPS		Incorrect
SMALL ULTRASOUND PROBE		Incorrect
ULTRASOUND EQUIPMENT		Incorrect
INTENSIVE CARE VENTILATOR		Incorrect
DRAINAGE BAGS		Incorrect
E.R. BAG		Incorrect
BLOOD 0+		Incorrect
BIG ULTRASOUND PROBE		Incorrect
CTG MONITOR		Incorrect
LISAcath(R)		Incorrect

Table S2. Partially correct/incorrect equipment items defined in DIANA game.
